# A Case of Stress-Induced Cardiomyopathy After Ketamine Infusion

**DOI:** 10.7759/cureus.59709

**Published:** 2024-05-05

**Authors:** Zach Hart, Thomas Anderson, Hanna Fanous, Sotiria Liori, Spencer Carter

**Affiliations:** 1 Internal Medicine, University of Utah Health, Salt Lake City, USA; 2 Cardiovascular Medicine, University of Utah Health, Salt Lake City, USA

**Keywords:** cardiac imaging-mri, catecholamine-induced myocardial toxicity, iv ketamine, heart failure with reduced ejection fraction, stress induced cardiomyopathy

## Abstract

We report the case of a 64-year-old female with a history of hypothyroidism and isolated parotid sarcoidosis who presented with acute-onset chest pain and dyspnea. Echocardiogram demonstrated transiently reduced ejection fraction with apical hypokinesis, without evidence of obstructive coronary lesions on angiography, compatible with stress-induced cardiomyopathy. She received a ketamine infusion as a mental health treatment shortly before the development of symptoms, suggesting that this medication may have precipitated her cardiomyopathy. In recent years, ketamine has become a popular option for treating mental health disorders, including major depressive disorder and substance use disorders. It should be used with caution in patients with known cardiovascular disease, and its cardiac effects warrant further study.

## Introduction

Stress-induced cardiomyopathy, also known as Takotsubo cardiomyopathy, is a clinical syndrome characterized by heart failure symptoms, electrocardiogram (EKG) changes, and biochemical markers suggestive of ischemia with no coronary obstruction in the absence of pheochromocytoma and myocarditis. Transient left ventricular apical hypo-/akinesis with relative preservation of function of the basal portion is common, though other patterns are increasingly recognized. Wall motion abnormalities classically extend beyond a single coronary distribution. This syndrome most commonly affects post-menopausal females, and cardiac function typically returns to normal [1,2]. The etiology of stress-induced cardiomyopathy has not been completely elucidated, but an excess of catecholamines is believed to play a role [1,2].

Ketamine is a non-competitive antagonist and allosteric modulator of N-methyl-D-aspartate (NMDA) receptors. It has long been used as an anesthetic. More recently, its use has expanded in treating psychiatric conditions, including major depressive disorder, schizophrenia, and substance use disorders. Data is limited regarding the cardiovascular effects of this medication. However, it is known to increase sympathetic activity by inhibiting neuronal catecholamine uptake and stimulating noradrenergic neurons to increase norepinephrine release into the bloodstream [3].

## Case presentation

A 64-year-old female with a past medical history of hypothyroidism and parotid sarcoidosis with no active disease presented to the emergency department with acute-onset left-sided chest pain, dyspnea, and orthopnea one hour after receiving her first ketamine infusion for "depression." Past medical history was notable for well-controlled hypothyroidism and sarcoidosis diagnosed by parotid biopsy 15 years ago without recurrence. Family cardiac history included myocardial infarction in her paternal grandfather and uncle. Medications included liothyronine, phentermine, semaglutide for weight loss, quetiapine, trazodone, progesterone, estradiol, dehydroepiandrosterone, and oxycodone. She had a distant history of methamphetamine and cocaine use and had been in remission for 14 years. She had one drink of alcohol per day. She used psychedelic mushrooms and lysergic acid diethylamide (LSD) once per month.

**Figure 1:**
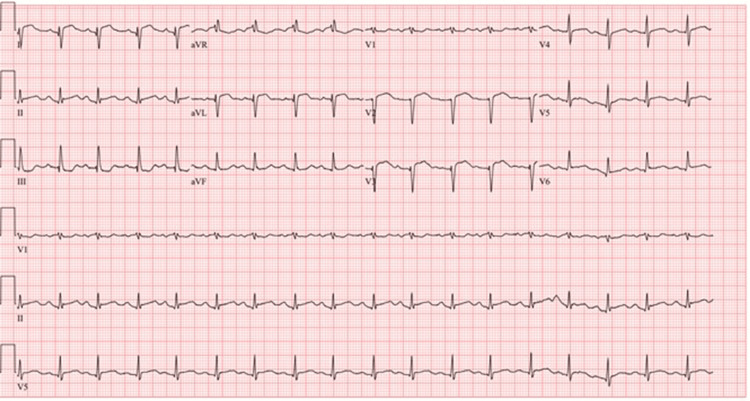
EKG at presentation EKG showed a sinus tachycardia with ST elevations in the antero-lateral leads and reciprocal ST depressions concerning for occlusive myocardial infarction EKG: electrocardiogram

**Figure 2:**
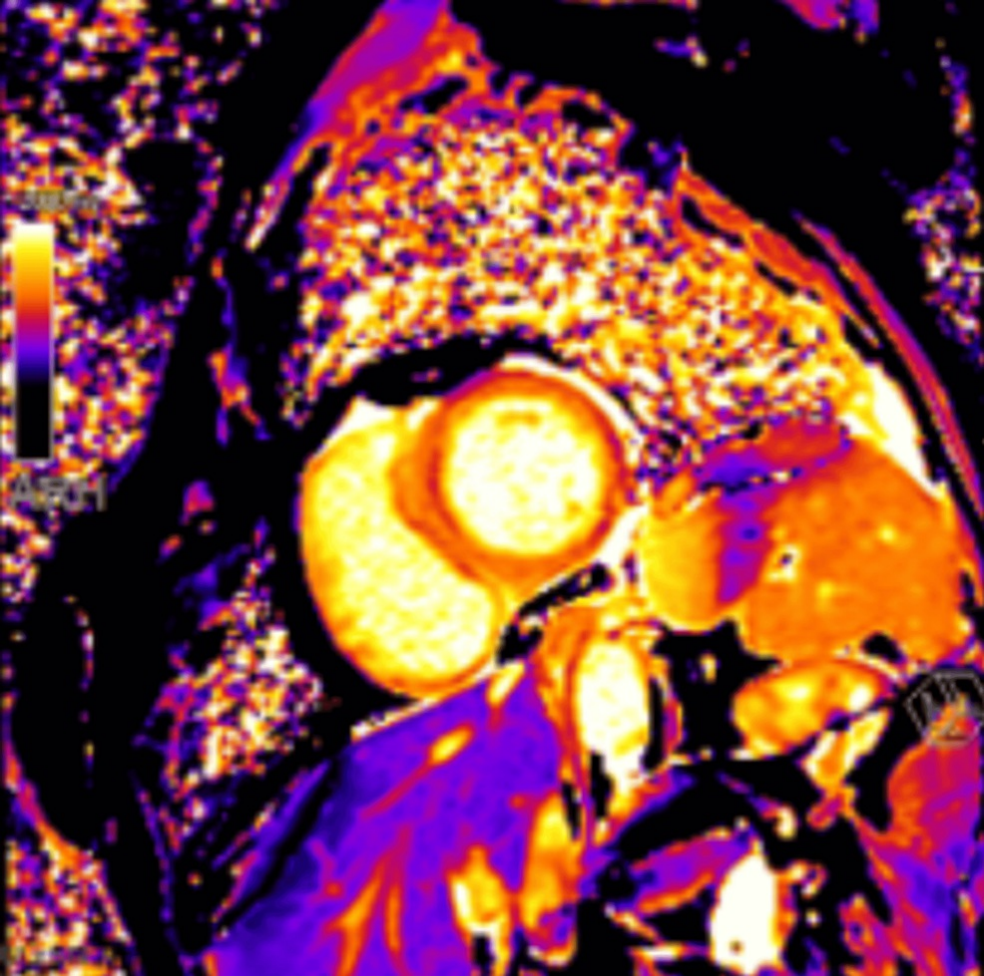
Cardiac MRI parametric mapping on 3T scanner Study was not suggestive of myocarditis or sarcoidosis. Native T1 elevated at 1456 ms (normal ≤1290 ms), ECV 33% (normal ≤32%) ECV: external cephalic version

She presented with sinus tachycardia to 130, hypotension with a narrow pulse pressure, and tachypnea with low normal oxygen saturation. Her jugular venous pressure was elevated to 13 cm, and crackles were heard at bilateral lung bases. Point-of-care ultrasound revealed reduced systolic function with apical hypokinesis and basal hyperkinesis. Initial laboratory work revealed a leukocytosis of 14,000 cells/μL with a left shift. Complete blood count (CBC) and comprehensive metabolic panel (CMP) were otherwise unremarkable. Troponin I was 3.89 ng/mL and peaked at 5.97 ng/mL three hours later. A respiratory panel, HIV testing, thyroid-stimulating hormone (TSH), erythrocyte sedimentation rate (ESR), and C-reactive protein (CRP) were within normal limits. Urine drug screen was positive for oxycodone and stimulants (presumed from phentermine use) but negative for other opiates and all other substances tested. Specific urine testing for amphetamines was negative, suggesting the positive stimulant test was not methamphetamine. Electrocardiogram showed sinus tachycardia with ST elevations in the antero-lateral leads and reciprocal ST depressions concerning for occlusive myocardial infarction (Figure 1). She was treated with aspirin, ticagrelor, heparin, and sublingual nitroglycerin with improvement of her chest pain. An emergent left heart catheterization revealed angiographically normal epicardial coronary arteries, sluggish coronary blood flow, and a left ventricular end-diastolic pressure of 29 mmHg (Video 1). CT angiogram revealed pulmonary interstitial edema but no pulmonary embolus. Transthoracic echocardiogram showed an ejection fraction of 21%, basal hyperkinesis with mid to apical akinesis of the left ventricle, and reduced right ventricular systolic function with severe hypokinesis of the mid and apical segment (Video 2). Cardiac MRI on a 3T scanner with and without contrast revealed an ejection fraction of 22% with apical hypokinesis and basal sparing. A small amount of late gadolinium enhancement (LGE) was seen at the right ventricular insertion, with no LGE elsewhere. Parametric mapping was completed with an elevated native myocardial T1 relaxation time of 1456 ms (normal ≤1290 ms) and extracellular volume fraction of 33% (normal ≤32%) (Figure 2). The study was not consistent with myocarditis or sarcoidosis.

**Video 1:** Coronary angiography results Left heart catheterization obtained after initial concern for acute coronary syndrome reveals angiographically normal epicardial coronary arteries with sluggish coronary blood flow

**Video 2:** EKG at presentation Transthoracic EKG showing a left ventricular ejection fraction of 21% with apical hypokinesis with relative basal hyperkinesis EKG: echocardiogram

Given her chest pain and EKG changes, this case was initially felt to represent an acute myocardial infarction. However, the reassuring coronary angiogram and characteristic echocardiogram led to the diagnosis of stress-induced cardiomyopathy. Myocarditis and cardiac sarcoidosis were considered as alternative diagnosis, but the cardiac MRI was not suggestive of either condition. Furthermore, she did not have a restrictive cardiomyopathy, which would have been more likely with sarcoidosis. 

She was admitted to the cardiovascular ICU and treated with intravenous diuretics. She was initially treated with doses of furosemide 80 mg IV with suboptimal urine output. Urine output improved the following day with two doses of bumetanide 6 mg IV, but she remained tachycardic with a narrow pulse pressure 34 hours after admission. After the echocardiogram showed no left ventricular outflow obstruction, she was started on a continuous dobutamine infusion at 3 mcg/kg/hr. She continued to receive this medication for three days with gradual weaning of the dosage and normalization of vital signs. She was transferred to the floor heart failure service and began guideline-directed medical therapy. She was discharged home on dapagliflozin, spironolactone, and losartan after an eight-day hospital stay.

The patient was seen in the cardiology clinic three weeks after discharge. She continued to have dyspnea while walking up steep hills but was able to complete 30 minutes of aerobic activity. She had no lower extremity edema or orthopnea. Repeat echocardiogram showed the normalization of ejection fraction and wall motion.

## Discussion

Our patient's acute-onset heart failure, presentation concerning for ACS, normal coronary arteries, and typical transient echocardiographic findings support the diagnosis of stress-induced cardiomyopathy. While the pathophysiology of stress-induced cardiomyopathy is incompletely understood, an excess of catecholamines is thought to play a central role [1,2]. However, the mechanism by which catecholamines and sympathetic activity cause cardiotoxicity is complex, and there are other factors that appear to influence this disease entity [2,4]. Microvascular dysfunction, metabolic abnormalities including in myocardial glucose metabolism, estrogen deficiency, and inflammation are other proposed etiologies of stress-induced cardiomyopathy [2,4].

Ketamine is known to increase both peripheral and central catecholamine levels via central sympathetic stimulation and inhibition of neuronal catecholamine reuptake, causing hypertension and tachycardia [3,5,6]. This pharmacologic profile aligns with the catecholamine hypothesis of stress-induced cardiomyopathy. While data is limited, the American Heart Association has listed ketamine as a medication that may exacerbate known heart failure [7]. This claim is supported by small, randomized studies showing worsening of cardiac function in patients with heart failure treated with ketamine [8]. In two distinct cases, ketamine administration has been associated with stress-induced cardiomyopathy. The first case reported it in a patient receiving ketamine alongside epinephrine for status asthmaticus [9]. More recently, a case report showed stress-induced cardiomyopathy in a patient following ketamine administration for a distal radius fracture reduction [10]. Both cases underscore potential cardiac risks associated with ketamine.

Given the temporal relationship between receiving ketamine and acute-onset heart failure, we believe our patient developed stress-induced cardiomyopathy secondary to ketamine infusion. While other potential triggers exist for our patient including estrogen deficiency and chronic phentermine use, the immediate onset of symptoms following ketamine infusion is striking. It is possible these factors placed her at a higher risk for developing stress cardiomyopathy when exposed to a precipitant. Notably, cardiac MRI was not suggestive of sarcoidosis or myocarditis.

## Conclusions

Stress-induced cardiomyopathy should be considered as a diagnosis in patients presenting with anginal chest pain, symptoms of heart failure, and wall motion abnormalities that extend beyond a coronary distribution, especially if catheterization reveals non-obstructive coronary arteries. There are many potential triggers for this condition, and medications, especially those that increase catecholamine levels, should be considered. Ketamine, one such medication, should be used with caution in patients with heart failure. This caution should likely be extended to those with a history of stress-induced cardiomyopathy. In fact, other options for treating mental health conditions should likely be chosen in this patient population when at all possible. As ketamine infusion becomes more prevalent in addressing mental health conditions, attention should be paid to the rates of stress-induced cardiomyopathy surrounding its use, especially in patients without a known history of heart failure. Further study investigating the cardiovascular effects of ketamine is warranted.
